# Disassembly of the cystovirus ϕ6 envelope by montmorillonite clay

**DOI:** 10.1002/mbo3.148

**Published:** 2013-12-19

**Authors:** Karin A Block, Adrianna Trusiak, Al Katz, Paul Gottlieb, Alexandra Alimova, Hui Wei, Jorge Morales, William J Rice, Jeffrey C Steiner

**Affiliations:** 1 Department of Earth and Atmospheric Sciences, City College of New York160 Convent Avenue, New York, NY, 10031; 2 Department of Physics, City College of New York160 Convent Avenue, New York, NY, 10031; 3 Sophie Davis School of Biomedical Education, City College of New York160 Convent Avenue, New York, NY, 10031; 4 Department of Biology, City College of New York160 Convent Avenue, New York, NY, 10031; 5 New York Structural Biology Center89 Convent Avenue, New York, NY, 10031

**Keywords:** Viral envelope disassembly, viral infectivity, virus adsorption, virus–montmorillonite heteroaggregation, ϕ6 nucleocapsid.

## Abstract

Prior studies of clay–virus interactions have focused on the stability and infectivity of nonenveloped viruses, yielding contradictory results. We hypothesize that the surface charge distribution of the clay and virus envelope dictates how the components react and affect aggregation, viral stability, and infectivity. The bacteriophage *Cystoviridae* species φ6 used in this study is a good model for enveloped pathogens. The interaction between φ6 and montmorillonite (MMT) clay (the primary component of bentonite) is explored by transmission electron microscopy. The analyses show that MMT–φ6 mixtures undergo heteroaggregation, forming structures in which virtually all the virions are either sequestered between MMT platelet layers or attached to platelet edges. The virions swell and undergo disassembly resulting in partial or total envelope loss. Edge-attached viral envelopes distort to increase contact area with the positively charged platelet edges indicating that the virion surface is negatively charged. The nucleocapsid (NCs) remaining after envelope removal also exhibit distortion, in contrast to detergent-produced NCs which exhibit no distortion. This visually discernible disassembly is a mechanism for loss of infectivity previously unreported by studies of nonenveloped viruses. The MMT-mediated sequestration and disassembly result in reduced infectivity, suggesting that clays may reduce infectivity of enveloped pathogenic viruses in soils and sediments.

## Introduction

It is estimated that there are 10^31^ virus particles on Earth making viruses more prevalent in number than prokaryotes, the vast majority of viruses being bacteriophages (Weinbauer 2004; Breitbart and Rohwer 2005). Clays are a primary nonorganic component of soils and aquatic sediments, therefore the dynamic interactions between clay minerals and phage are expected to affect soil–bacteria activity (Ostle and Holt 1979; Vettori et al. 1999; Weinbauer and Rassoulzadegan 2004; Syngouna and Chrysikopoulos 2010). Montmorillonite (MMT) is a highly reactive, expandable, hydrous aluminum smectite clay. Smectite platelets are positively charged at the edges and negatively charged along the faces to produce an overall negative charge at pH > 3. Clays readily form colloidal suspensions that have the potential to interact with dispersed viral particles which influences the aggregate structure.

Clay speciation is important in considering the fate of viral particles in the environment. Numerous studies on the interaction between viruses and clays have yielded conflicting information regarding the effect of clay minerals on virus survival and infectivity; see Jin and Flury (2002) or Kimura et al. (2008) for reviews. The contradictory findings in the literature imply that virus morphology plays a role in the interaction with clays. To our knowledge, published studies on the interactions between clays and viruses have only investigated nonenveloped viruses, although a number of pathogenic viruses found in the environment are enveloped (e.g., avian influenza, coronavirus). Enveloped viruses differ from nonenveloped viruses in that they possess a lipid–protein layer surrounding the nucleocapsid (NC). *Cystoviridae*, a bacteriophage family that infects the plant pathogen, *Pseudomonas syringae* pv. *phaseolicola*, is one of the few phage families with an external lipid envelope. *Cystoviridae* species are often used as a model for enveloped animal and human pathogens (Mindich 1988, 2004).

Many studies have focused on clay influence on virus survivability, but only several have investigated factors that can affect the virus–clay interaction. Roper and Marshall (1974) found that at low salinity, MMT particles surrounded *Escherichia coli* bacteria resulting in greater protection from phage than under higher salinity conditions. Roper and Marshall (1978) determined that MMT inhibited infection of *E. coli* and that finer suspended clay particles provided the *E. coli* with greater protection from phage by forming a barrier around the bacteria. However, they found that particles greater than 0.6 *μ*m in diameter offered no protection. Lipson and Stotzky (1984) observed that several proteins (lysozyme, chymotrypsin, and ovalbumin) reduced the adsorption of reoviruses to MMT, likely a result of the proteins competing for adsorption sites on the clay. In contrast, kaolinite and MMT enhanced *φ*X174 infection of *E. coli* (Lipson and Alsmadi 1989). Zeph et al. (1988) determined that MMT protected phage P1 from inactivation, although the presence of MMT had no effect on transduction of *E. coli*. Vettori et al. (2000) found that clay minerals protect phage PBS1 from inactivation and loss of ability to transduce *Bacillus subtilis* by UV light.

Animal pathogenic viruses similarly interact with clays. Vilker et al. (1983a,1983b) investigated the interaction of poliovirus with nonaggregated MMT, and through scanning electron microscope (SEM) analysis determined that the negatively charged virions adhere to the positively charged MMT edges. They also suggested that clay aggregation accompanies adsorption of poliovirus and that the MMT enhances poliovirus survival (Vilker et al. 1983a,1983b). Lipson and Stotzky (1983, 1986) showed that viruses (e.g., poliovirus, coxsackie virus, reovirus) are adsorbed onto clays with enhanced survivability. The adsorption of reovirus onto MMT or kaolinite was almost immediate and correlated with the cation exchange capacity of the clays (Lipson and Stotzky 1983). This indicates that reovirus adsorbs onto negatively charged regions of the clays. Lipson and Stotzky (1985) found that coliphage T1 and reovirus type 3 adsorb onto different sites on kaolinite and MMT. They also investigated mixtures of kaolinite and reovirus and determined that these were more infectious than equivalent concentrations of virus alone and attributed the increased infectivity to improved viral transport in the presence of kaolinite.

The seemingly contradictory effects of clays on virus survivability are likely a result of differences in the mechanisms behind the clay–virus interactions. Although the literature has focused on the effect of environmental conditions (clay type, clay charge distribution [Lipson and Stotzky 1985; Christian et al. 2006; Templeton et al. 2008], pH [Zhuang and Jin 2008; Walshe et al. 2010], ionic strength [Tong et al. 2012], buffer composition [Zhuang and Jin 2008; Gutierrez et al. 2010], and cation exchange capacity [Lipson and Stotzky 1983; Vettori et al. 1999]), the surface morphology of viruses has been largely ignored. The attachment of phages T1, T7, T2, PBS1, or *φ*X174 to cation exchanged clay is related to positively charged sites on clay edges (Schiffenbauer and Stotzky 1982; Chattopadhyay and Puls 1999; Vettori et al. 2000). However, Lipson and Stotzky (1983) found that reovirus adsorption occurs at negatively charged sites on cation exchanged kaolinite and MMT, consistent with Derjaguin, Landau, Verwey, Overbeek (DLVO) (Derjaguin and Landau 1941; Verwey and Overbeek 1948) theory of colloidal aggregation. Rossi and Aragno (1999) found that colloidal aggregation of phage T7 and MMT results in reversible binding and protection from inactivation. Phages MS2 and *φ*X174 attach to kaolinite and MMT by hydrophobic interactions (Chrysikopoulos and Syngouna 2012). Chattopadhyay and Puls (2000) studied the different forces involved in phage (T2, MS2, *φ*X174) sorption on soil particles (hectorite, kaolinite, and Norman clay) and found that van der Waals attraction dominated over electrostatic repulsion.

In this work, the objective is to explore the interaction between MMT clay and an enveloped virus to better understand the mechanisms of MMT-induced virus deactivation in clay-rich environment, such as Earth's critical zone. In being the first study of the interaction between clays and enveloped viruses, the aim is to provide insight into the methods of viral inactivation, and the application to many enveloped human pathogens.

## Material and Methods

### Montmorillonite clay

A high-purity Na-MMT (smectite) clay (commercial name: “Accofloc”; chemical formula: (Na,Ca)_0.33_(Al_1.67_Mg_0.33_)Si_4_O_10_(OH)_2_ n(H_2_O); American Colloid Company, Arlington Heights, IL) was made homoionic with magnesium using the cation substitution technique described by Moore and Reynolds (1997). The clay was initially washed in 5% sodium hypochlorite (bleach) to remove organic contaminants and then washed multiple times in distilled water to eliminate the bleach. Large clay particles and nonclay minerals were removed by centrifugation at 3600 rpm (2700*g*) for 20 min. The supernatant comprising the fraction with an equivalent spherical Stokes diameter less than 0.2 *μ*m was collected for use in this experiment. The purified MMT was suspended in 0.1 mol/L MgCl_2_ overnight and centrifuged at 1300 rpm (350*g*) for 30 min. The pellet was rinsed in distilled water 8–10 times and resuspended in distilled water. Two drops of AgNO_3_ were added to the suspension after the rinses to verify that all chloride was removed. The concentration (w/v) of the stock suspension was 2 mg mL^−1^. The clay suspension was autoclaved just prior to addition of the viruses to ensure sterility.

### Purification of ϕ6

φ6 is the first isolated member of the *Cystoviridae* family. The φ6 virion has a layered structure consisting of an inner NC surrounded by the lipid envelope (Kenney et al. 1992). The φ6 bacteriophage host cell, *P. syringae* LM2333 growth medium is Luria Bertani, supplemented with ampicillin (100 *μ*g/mL) to inhibit contamination. Buffers A and ACN were used for the suspensions of purified φ6. Buffer A contains 10 mmol/L KH_2_PO_4_ (pH 7.5) and 1 mmol/L MgSO_4_. Buffer ACN is buffer A with the addition of 200 mmol/L NaCl and 0.5 mmol/L CaCl_2_. Plate lysates of φ6 were prepared by plating phage dilutions into soft agar with a culture of LM2333. The plates were incubated overnight at room temperature prior to collecting the phage-containing top agar. The cell debris and agar were removed by centrifugation in a Sorvall GSA rotor (Thermo Scientific, Asheville, NC) at 15,000 rpm (3.0 × 10^4^*g*) for 30 min at 4°C. Virus was collected by centrifugation in a Beckman T-1270 rotor at 33,000 rpm (7.5 × 10^4^*g*) for 2 h at 4°C. The pellet was suspended in 1 mL of buffer A. The bacteriophage samples were then layered on a 10–30% sucrose gradient in buffer A. Sedimentation centrifugation was at 23,000 rpm (6.7 × 10^4^*g*) for 1 h at 15°C using a Beckman SW 40 Ti rotor, after which the virus particle band was visualized by light scattering and the band collected by needle puncture, pelleted by centrifugation at 33,000 rpm (7.5 × 10^4^*g*) for 2 h at 4°C, and resuspended in 1 mL of buffer A. Final purification of the phage was by equilibrium centrifugation through 40–60% sucrose gradient in buffer A using a Beckman SW 40Ti rotor at 23,000 rpm (6.7 × 10^4^*g*) overnight at 4°C. The next day the phage band was again visualized by light scattering and collected by tube puncture. The phage sample was then centrifuged with a Sorvall T-1270 rotor at 33,000 rpm (7.5 × 10^4^*g*) for 2 h at 4°C and the collected phage particles suspended in 100 *μ*L buffer ACN.

Turbidity measurements of φ6 following the procedure described by Oster (1951) demonstrated that φ6 surface charge is negative at pH 7 (spectra not shown).

### Purification of NC

The NC particles were isolated by removing the φ6 envelope with Triton X-100 as described by Steely and Lang (1984). The purified φ6 virus was mixed with 10% Triton X-100 solution in buffer ACN. ACN buffer is recommended because of the presence of Ca^2+^ ions which stabilize the NC particles, preventing the loss of P8 proteins. The particles were centrifuged at 33,000 rpm (7.5 × 10^4^*g*) for 1.5 h at 4°C. Pellets were washed and resuspended in ACN buffer. Although NC concentrations cannot be verified by plaque formation, triton X-100 envelope removal is essential complete and thus the NC concentrations are approximately equal to the initial whole-virion concentrations.

### Montmorillonite–virus mixtures

Sterilized Mg-MMT (500 *μ*L) suspensions were mixed in Eppendorf tubes with 100 *μ*L φ6 bacteriophage in ACN buffer. Tris buffer (10 *μ*L) was added to bring the resultant mixture to 0.5 mmol/L CaCl_2_ and 1 mmol/L MgSO_4_. NC-MMT mixtures were prepared in the same manner (500 *μ*L of MMT, 100 *μ*L of NCs in ACN buffer and Tris). The concentration of cations in the buffer was sufficient to initiate fast aggregation of MMT (Katz et al. 2013). Visual inspection confirmed that the mixture aggregated in less than a few minutes. φ6 in ACN buffer with Tris, but without MMT was used as a control. The Eppendorf tubes were incubated at room temperature (25°C) for an hour. The mixtures were then centrifuged for 10 min at 3600 rpm (1200*g*). The supernatant was collected and the clay pellet resuspended in 100 *μ*L ACN buffer. The pelleted aggregates were vortexed prior to plaquing to disaggregate the mixture and separate the viruses from the MMT. The supernatants and final, disaggregated, clay suspensions were spot checked on plates with growing *P. syringae* to determine fractionation of virus in the system. Virus concentrations were calculated using the plaque assay technique. The NC populations used in the transmission electron microscopy (TEM) analysis were made from comparable φ6 concentrations. Phage infectivity was measured over a range of 6 orders of magnitude of MMT-to-phage ratios (mg of MMT/number of virions).

### Transmission electron microscopy

Samples were negatively stained with uranyl acetate. A minimal amount of stain was employed to reduce the effect of the stain on abundant viral proteins, which would impede identification of viral particles. TEM grids were prepared using φ6 concentrations of 10^11^ virions mL^−1^ and MMT concentration of 1.7 mg mL^−1^. Electron micrographs were acquired at the New York Structural Biology Center with either a Jeol 2100 (Jeol Inc., Peabody, MA) or a Tecnai FEI 20 (FEI, Hillsborough, OR) electron microscope, both operating at 200 kV. For the Jeol 2100, micrographs were acquired at magnifications of 30,000× or 50,000× giving pixel sizes of 0.511 or 0.306 nm/pixel, respectively, on the 2k × 2k CCD. For the Tecnai F20, micrographs were acquired at magnifications of 29,000× or 50,000× giving pixel sizes of 0.592 or 0.340 nm/pixel, respectively, after 2 × 2 binning of the 4k × 4k CCD. Isolated φ6 virions and NCs are nearly spherical in shape and when negatively stained will appear in electron micrographs as ˜70 nm and ˜57 nm circles, respectively. φ6 virions have a clearly defined envelope that appears as a lower density region under negative staining. The NC outer icosahedral shell also appears as a brighter region in the micrographs when negatively stained. Classification of intact φ6 virions and MMT-induced, enveloped-stripped NCs (MMT-NC) was determined by size and shape analysis. Particles exhibiting nearly circular shape in the micrographs with a diameter between 68 nm and 75 nm were classified as nondistorted φ6. Nearly circular particles with diameters between 55 and 63 nm diameters were classified as nondistorted MMT-NCs. Virions deviating from circularity were classified as complete but distorted φ6 if the larger diameter was greater than 68 nm, or distorted MMT-NCs if the larger diameter was less than 63 nm.

Size distributions of the isolated φ6 and NCs were calculated using the particle analysis function of ImageJ software (U. S. National Institutes of Health, Bethesda, MD; Schneider et al. 2012). Poor contrast between embedded virions and MMT hinders the ability to use the automatic particle size and counting functionality in standard image processing software. Therefore, size analysis was performed by counting pixels and converting to distance using the calibrated pixel size of the microscopes. CorelDraw was used to resample the insets shown in Figure 3 at twice the pixel density, and autoadjust the contrast.

## Results and Discussion

### TEM of phage φ6, NC, and montmorillonite

**Figure 1:**
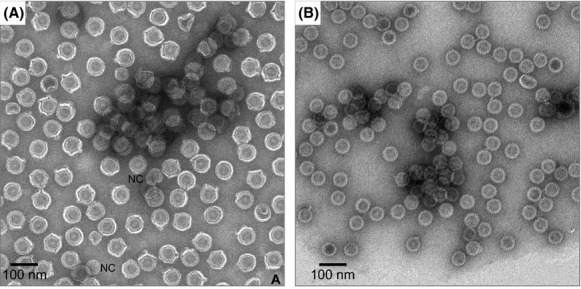
TEM micrographs of (A) φ6 and (B) NCs with trace amounts of MMT. φ6 and NC particles appear consistently round. Only two NCs are identifiable in (A) (labeled NC) in a field of 10^2^ φ6 virions.

TEM micrographs of φ6 and NC in Figure 1A and B, respectively, reveal a consistency of particle size in the absence of clay that is not affected by uranyl acetate staining. The micrograph in Figure 1A shows ˜10^2^ φ6 virions with diameter 69 ± 2 nm for over 95% of the particles. The vast majority of the virions in Figure 1A exhibit aspect ratios near unity (1.08 ± 0.07), confirming the spherical morphology of the particles. Only two NC particles with diameters of 57 nm (labeled NC in Fig. 1A) are present in the field of view, confirming that the φ6 envelope rarely spontaneously disassembles. Very few partially disassembled φ6 are visible in the micrograph. Although NC particles are icosahedral, the NC particles appear spherical at the resolution of the Figure 1B micrograph. The vast majority of the NCs in Figure 1B have a diameter of 57 ± 2 nm and aspect ratios near unity (1.05 ± 0.02). Therefore, size and aspect ratio clearly distinguish intact φ6 and NCs and can be used to gauge morphological changes in the virions as a result of interactions with suspended MMT.

**Figure 2:**
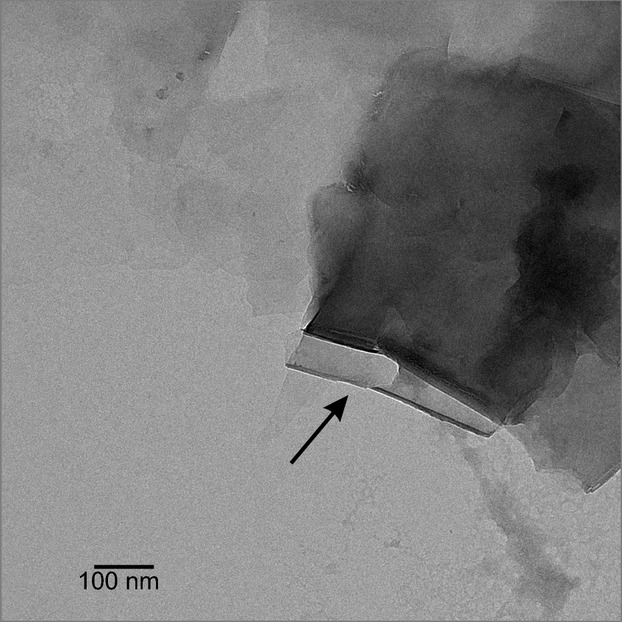
A typical TEM micrograph of MMT platelets of varying thickness and relative orientation showing lack of features on a size scale of viral particles. The edge view (arrow) is indicative of platelet stacking.

Figure 2 shows a representative TEM micrograph of MMT platelets. The clay platelets exhibit a sheet-like structure (platelet face) which in the TEM aggregate results in a mosaic of sharp angular forms with discrete edges. The arrow in Figure 2 indicates parallel edges which is indicative of platelet stacking. The platelet faces exhibit a mottled texture and lack geometric characteristics similar to those of viral particles.

### TEM of φ6–montmorillonite aggregates

An important characteristic of the micrographs is the complete absence of either free virions or virion-only aggregates at sufficient MMT concentrations to produce visible aggregates (˜1 mg mL^−1^). In all the TEM grids, the entire φ6 phage population is sequestered within aggregates. A majority of the virions are sandwiched between platelets and partially obscured due to masking by thin MMT sheets. However, a noticeable fraction of the φ6 are attached to the edges of the platelets and are clearly discernible in the micrographs. A preponderance of edge-attached and face-attached virions exhibits extensive distortion in shape, in contrast to the uniformity of particles shown in Figure 1A and B. The presence of smaller (˜60-nm-diameter) particles in the micrographs is interpreted as clear evidence of disassembled envelopes leaving only NCs. Approximately third of the φ6 in the micrographs are completely stripped of their envelopes and are morphologically similar in size to distorted NC particles. The microscopy evidence therefore reveals that the interaction with MMT results in the disassembly of the φ6 envelope.

**Figure 3:**
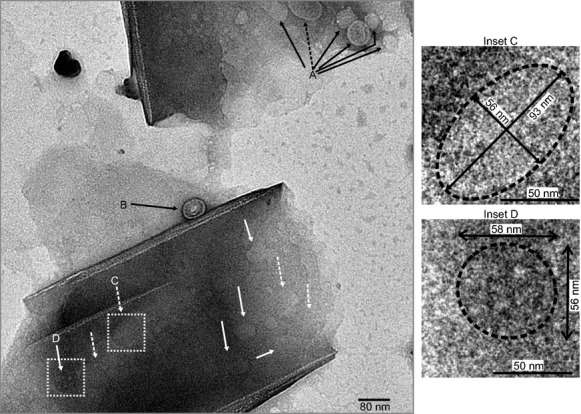
TEM micrograph showing a population of virions in two clay particles in differing states of disassembly associated with two MMT particles. A population of virions attached to platelet surface is labeled A. Particle labeled B is an edge-attached virion with a disassembled envelope. Dashed arrows indicate distorted or partially disassembled virions; solid arrows point to virions that have undergone total envelope removal leaving only the NCs. Particles labeled C (distorted φ6) and D (φ6 with envelope disassembled) are shown in respective insets.

Cation bridging between the negatively charged platelet faces and negatively surface-charged viruses facilitates a van der Waals attraction causing virions to attach to platelet faces. This attraction is consistent with DLVO theory of colloidal aggregation. The attachment of virions to the positively charged edges likely includes both electrostatic and van der Waals interactions. A TEM micrograph (Fig. 3) shows a population of φ6 in varying states of disassembly, and embedded in two well-defined clay particles. Top platelet – a distorted φ6 virion (dashed arrow) and several MMT–NCs (solid arrows) appear embedded within layers of clay at position labeled A. The particles near the platelet edge (far upper right) are distinctly smaller than triton-isolated NCs indicating partial disassembly. Bottom platelet – a distorted 57 nm MMT-NC attached to the clay edge is evident (particle labeled B). The MMT-NC distortion increases the contact area between the particle and the positively charged MMT edge, suggesting that the outer surface of the P8 protein shell acquired a negative charge during disassembly. A number of distorted φ6 (four are noted by dashed arrows) and MMT-NCs (five are noted by solid arrows) are located between platelets. A highly distorted virion (particle labeled C) is shown in inset C; particle size is 93 × 56 nm, corresponding to an aspect ratio of 1.7. The deformed virions indicate that either the envelope disassembly increases particle size as water infiltrates the lipid–protein membrane or compression of virions between platelets has caused distortion. The particle labeled D and shown in inset is nearly circular with a diameter ˜57 ± 1 nm indicating that the envelope has been completely removed.

**Figure 4:**
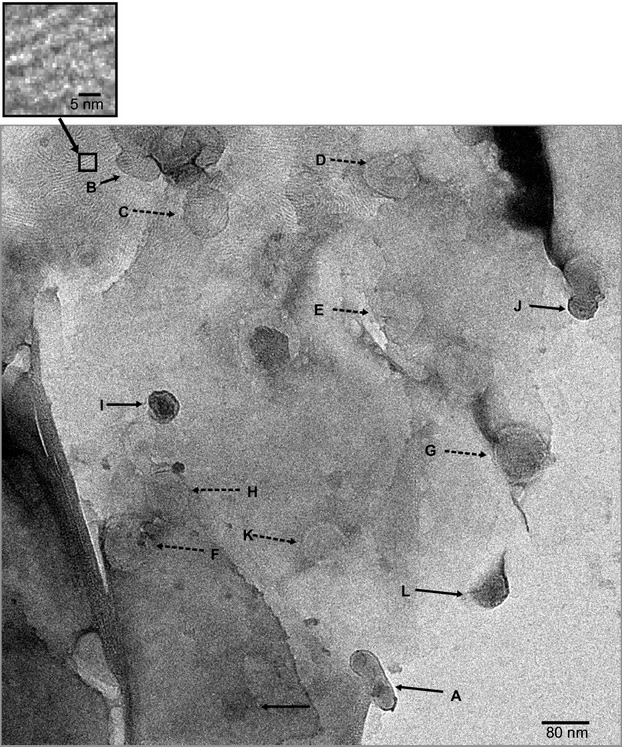
TEM micrograph of assemblies of virions in varying states of disassembly. Dashed arrows indicate distorted or partially disassembled virions; solid arrows point to virions that have undergone total envelope removal leaving only the NCs. Elongated particle labeled A is a fused virion. Inset shows alternating tetrahedral and octahedral layers in the clay indicative of edge-on orientation. Note that several virions are congregated near platelet edges at upper left of micrograph.

Figure 4 shows an aggregate of clay platelets and partially disassembled virions (dashed arrows) and MMT-NCs (solid arrows). A pair of fused virions is observed in the micrograph (the elongated shape labeled A, at the bottom center). Distorted φ6 and MMT-NCs are congregated near platelet edges (upper left of micrograph, labeled B, C, and D), further evidence that φ6 particles are sequestered in the MMT aggregates. Other virions with disrupted envelopes (E, F, G, H, and K), as well as deformed MMT-NCs (I, J, and L), are readily observable. The magnified inset depicts the repeating tetrahedral–octahedral MMT layering, visible when platelets are viewed edge-on. In this view, virions are shown to preferentially aggregate along the positive edges and wedged between vertically oriented platelets.

**Figure 5:**
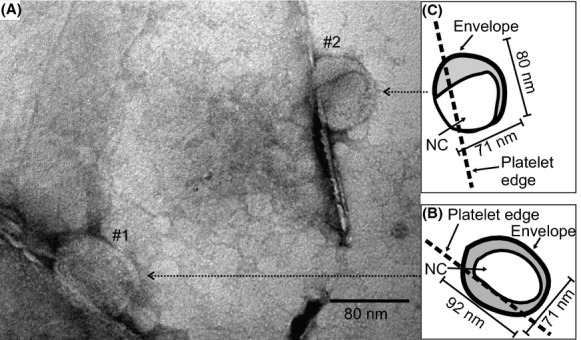
(A) TEM micrograph showing two φ6 virions attached to MMT platelet edges. The virions are distorted resulting in increased contact with the platelet edge. Outlines of the virion envelopes and NCs are shown for particles labeled #1 and #2 in (B) and (C), respectively. Envelope distortions and partial disassembly is evident from the tendency toward ellipsoidal shape of the virions and NC and increased spacing between the NC and envelope.

φ6 adhered to MMT edges exhibits a distinct oval shape in which the major axis is parallel to the edges, increasing contact area between the virion and the platelet edges. The increased contact suggests that the positively charged edge of the MMT is electrostatically interacting with the negatively charged virus. Two such virions are seen in the TEM micrograph shown in Figure 5A. A tracing of the outer boundary and the internal MMT-NC of virions labeled #1 and #2 are presented in the respective insets, B and C, respectively. Virion #1 has a shorter axis normal to the platelet edge (71 nm), which corresponds to the diameter of an undistorted virion. However, the elongated axis stretches to 92 nm resulting in an aspect ratio of 1.3. In contrast to virions interlayered with platelets (Fig. 4), where the distortion may be due to either expansion in two or three dimensions, the departure from sphericity noted here includes a volume increase. The boundary layer that includes the envelope (bright border) between MMT-NC and the outer surface of the virion is of variable thickness, as much as 17 nm, compared to the 6 nm envelope thickness reported by Kenney et al. (1992), suggesting that water is infiltrating the virion. Virion #2, while not appreciably different in size than an undistorted virion, deviates significantly from a spherical form. The envelope of virion #2 at the platelet edge also appeared to have undergone disassembly. The thicker region between the envelope and MMT-NC at the upper part of #2 may have resulted primarily from distortion or water absorption between the envelope and NC. The MMT-NC in virion #2 departs dramatically from a spheroidal geometry.

### TEM of NC–montmorillonite aggregates

**Figure 6:**
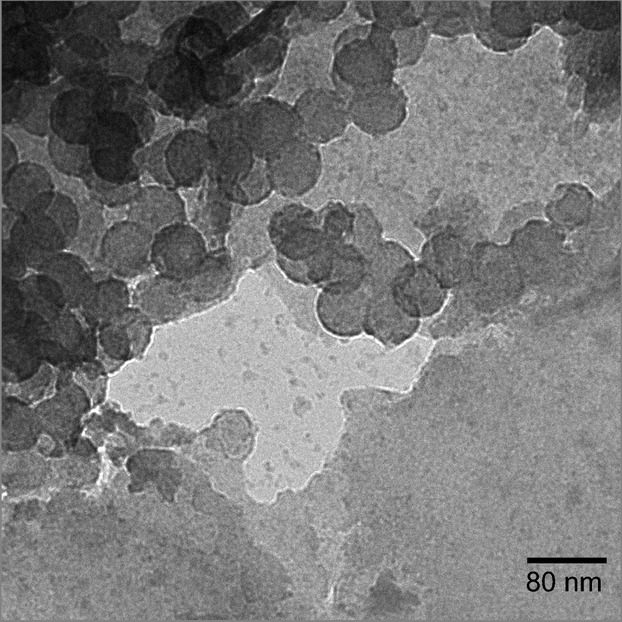
TEM micrograph showing detergent-produced NCs aggregated with MMT. The majority of NC particles in the aggregate exhibit little or no distortion and remain spherical with a diameter of ˜57 nm.

Mixing detergent-isolated NC particles with MMT creates aggregates that encompass virtually all the NC particles. The morphology of the NC particles in NC-MMT aggregates (TEM micrograph in Fig. 6.) appears similar to that of detergent-isolated NC particles. This is in distinct contrast to the MMT-NC particles produced by MMT-induced envelope disassembly. In the present case, the NC particles are roughly circular with a 57 nm diameter, that is, nearly identical in appearance to detergent-isolated NC particles. As the detergent-isolated NC particles in the aggregates retain their original morphology, the interaction with the clay is likely weaker than that between the MMT and the partially disassembled virions. This is consistent with the hydrophobic interactions reported for nonenveloped viruses (Chattopadhyay and Puls 1999, 2000; Chrysikopoulos and Syngouna 2012). It also suggests that Triton X100, which is nonionic, is less likely to produce negatively charged NCs in contrast to envelope-stripped MMT-NC particles produced by interaction with MMT.

### Sodium dodecyl sulfate polyacrylamide gel electrophoresis (SDS-PAGE)

**Figure 7:**
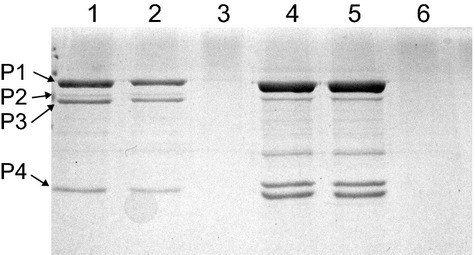
SDS-PAGE chromatograph showing the four larger molecular-weight proteins (P1, P2, P3, and P4) of phage φ6. The lanes are as follows: (1) isolated φ6 virions; (2) MMT–φ6 virion pellet; (3) MMT-φ6 virion supernatant isolated from pellet preparation; (4) isolated NC; (5) MMT-NC pellet; and (6) MMT-NC supernatant isolated from NC pellet preparation.

SDS-PAGE analysis was employed to confirm the TEM results that the φ6 and NCs were nearly completely sequestered in the MMT aggregates. An SDS-PAGE gel showing the larger molecular-weight φ6 proteins (P1, P2, P3, and P4) (Gottlieb et al. 1988) of φ6 and NCs is presented in Figure 7. Lane 1 is from purified φ6. Lane 2 is from the extracted pellet and lane 3 is from the supernatant of the φ6–MMT mixture. Virus concentration in the mixture was equal to that of purified φ6 in lane 1. Band intensities and migration positions in the pellet lane are comparable to those in the pure φ6 lane, whereas the φ6 proteins are almost entirely absent from the supernatant lane. This demonstrates that: (1) almost all the viral components remain sequestered in the MMT; and (2) the viral proteins are not being hydrolyzed by the MMT but remain intact. Lane 4 is from purified NC isolated by detergent. Effectiveness of NC isolation is confirmed by the absence of the P3 envelope protein band. Lane 5 is from the extracted pellet and lane 6 is from the supernatant of a mixture of MMT and detergent-isolated NC, also at an NC concentration equal to that used in lane 4. The comparable band intensities in lanes 5 and 6, in conjunction with the absence of NC protein bands in lane 6, demonstrate that the NC is also almost totally sequestered in the MMT aggregate without noticeable hydrolysis of viral proteins. These results are similar to those found for the whole-virus–MMT mixture. The absence of P3 in the NC lanes verifies that the NC preparation did not contain whole virus.

### Reduced φ6 infectivity by montmorillonite

**Figure 8:**
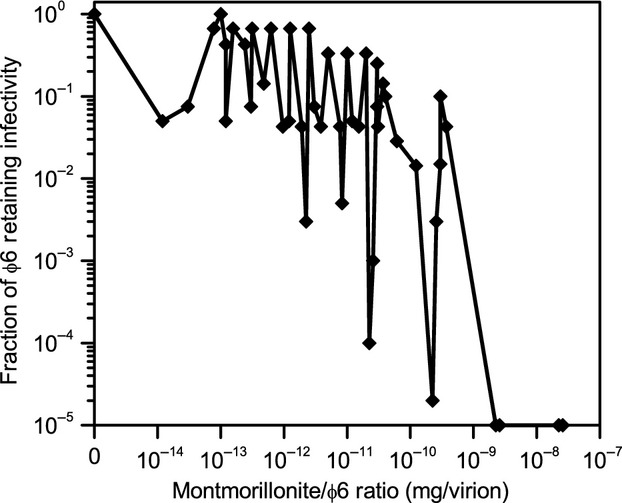
φ6 infectivity as a function of the MMT-to-φ6 ratio.

Plaque assays of the supernatant (assuming 100% infectivity) reveal that over the entire experimental series the fraction of φ6 that remained in the supernatant after centrifugation ranged from 10^−4^ to 10^−6^. In other words, more than 99.99% of the virions are in the pellet. This is in agreement with the TEM and SDS-PAGE analysis. Figure 8 plots the reduction in φ6 infectivity relative to a virus control (no MMT) for a range of ratios of MMT to φ6. For the 0.2 *μ*m MMT fraction used in these experiments, a MMT platelet has a mass of ˜10 fg, yielding about ˜10^11^ platelets mg^−1^. At virus concentrations exceeding the number of platelets, moderate loss of infectivity (20–95%) was observed. At virus concentrations lower than the number of platelets, infectivity is reduced by a factor of 10^−1.5^–10^−5^, where the least amount of loss of infectivity corresponded to smaller amounts of clay and the larger degree of loss corresponded to larger amounts of clay. The variation in infectivity loss at comparable platelet and virion populations (10^−9^–10^−11^ mg of MMT per virion) may be due to aggregation dynamics affecting the probability of virion–clay contact. This loss of infectivity resulted from either the partial disassembly or complete removal of the envelope. Since essentially all the φ6 aggregates with the MMT independently of virion concentration, the differences in infectivity under different relative concentration can be directly attributed to the probability of direct contact between virions and platelets. At relatively lower φ6 concentration, the probability that virions are in direct contact with platelets increases, resulting in greater envelope disassembly. However, at higher φ6 concentrations, many of the virions are more likely to only be in direct contact with other virions rather than platelets and thus less likely to experience envelope disassembly.

## Conclusions

Interactions between colloidal clay and suspended viral particles result in the stripping of the viral envelope and a consequent loss of infectivity of the phage φ6. As viral particles become embedded in growing clay aggregates, virions continue to be scavenged from the aqueous medium. TEM captures images of the φ6 virions in varying states of envelope disassembly. The extensive shape distortion exhibited by the edge-bound φ6 virions indicates that the forces are stronger than those binding the face-attached virions. This observation suggests that an electrostatic interaction is involved. The sandwiched virions are likely held preferentially by van der Waals forces to the negatively charged platelet faces as prescribed by DLVO theory. Both binding mechanisms contribute to the disassembly and swelling of virions and indicate that the envelope is pliable. The irreversible loss of infectivity through the process of envelope disassembly by MMT is a novel and previously unreported mechanism and is relevant to predicting the response of pathogenic enveloped viruses to clays in the environment.
